# Patterns of deer ked (Diptera: Hippoboscidae) and tick (Ixodida: Ixodidae) infestation on white-tailed deer (*Odocoileus virginianus*) in the eastern United States

**DOI:** 10.1186/s13071-021-05148-9

**Published:** 2022-01-20

**Authors:** Karen C. Poh, Jesse R. Evans, Michael J. Skvarla, Cody M. Kent, Pia U. Olafson, Graham J. Hickling, Jennifer M. Mullinax, Erika T. Machtinger

**Affiliations:** 1 Department of Entomology, Penn State University, University Park, PA 16802 USA; 2 Department of Environmental Science and Technology, University of Maryland, College Park, MD 20742 USA; 3 Livestock Arthropod Pests Research Unit, United States Department of Agriculture, Kerrville, TX 78028 USA; 4 The Center for Wildlife Health, University of Tennessee Institute of Agriculture, Knoxville, TN 37996 USA

**Keywords:** Deer keds, Ticks, White-tailed deer, *Odocoileus virginianus*, *Ixodes scapularis*, *Lipoptena cervi*, *Lipoptena mazamae*, Niche partitioning

## Abstract

**Background:**

White-tailed deer (*Odocoileus virginianus*) host numerous ectoparasitic species in the eastern USA, most notably various species of ticks and two species of deer keds. Several pathogens transmitted by ticks to humans and other animal hosts have also been found in deer keds. Little is known about the acquisition and potential for transmission of these pathogens by deer keds; however, tick-deer ked co-feeding transmission is one possible scenario. On-host localization of ticks and deer keds on white-tailed deer was evaluated across several geographical regions of the eastern US to define tick-deer ked spatial relationships on host deer, which may impact the vector-borne disease ecology of these ectoparasites.

**Methods:**

Ticks and deer keds were collected from hunter-harvested white-tailed deer from six states in the eastern US. Each deer was divided into three body sections, and each section was checked for 4 person-minutes. Differences in ectoparasite counts across body sections and/or states were evaluated using a Bayesian generalized mixed model.

**Results:**

A total of 168 white-tailed deer were inspected for ticks and deer keds across the study sites. Ticks (*n* = 1636) were collected from all surveyed states, with *Ixodes scapularis* (*n* = 1427) being the predominant species. Counts of *I. scapularis* from the head and front sections were greater than from the rear section. Neotropical deer keds (*Lipoptena mazamae*) from Alabama and Tennessee (*n* = 247) were more often found on the rear body section. European deer keds from Pennsylvania (all *Lipoptena cervi*, *n* = 314) were found on all body sections of deer.

**Conclusions:**

The distributions of ticks and deer keds on white-tailed deer were significantly different from each other, providing the first evidence of possible on-host niche partitioning of ticks and two geographically distinct deer ked species (*L. cervi* in the northeast and *L. mazamae* in the southeast). These differences in spatial distributions may have implications for acquisition and/or transmission of vector-borne pathogens and therefore warrant further study over a wider geographic range and longer time frame.

**Graphical Abstract:**

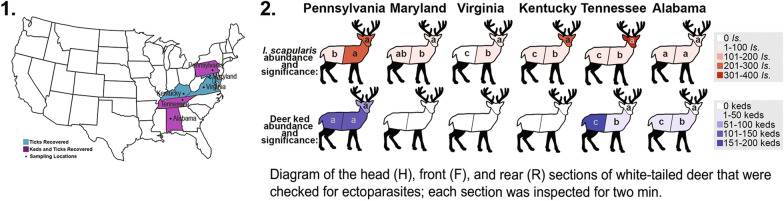

**Supplementary Information:**

The online version contains supplementary material available at 10.1186/s13071-021-05148-9.

## Introduction

White-tailed deer (*Odocoileus virginianus* (Zimmermann, 1780)) in the eastern US host numerous ectoparasites, including at least 19 species of ticks [1, 2] and 2 species of deer keds [3, 4]. These cervids are the principal host for adult blacklegged ticks (*Ixodes scapularis* Say, 1821) as well as winter ticks (*Dermacentor albipictus* (Packard, 1869)) and lone star ticks (*Amblyomma americanum* (Linnaeus, 1758)). Deer keds are hematophagous ectoparasitic flies, and the two species present in eastern North America are European deer keds (*Lipoptena cervi* (Linnaeus, 1758)) and Neotropical deer keds (*L. mazamae* Róndani, 1878). These two species are geographically distinct, with *L. cervi* found in the northeastern US and adjacent Canada and *L. mazamae* found in the southeastern US into South America, with a small region of range overlap in the Appalachian Mountains of western Virginia and eastern Tennessee [5–7].

As vector-borne disease cases continue to rise in the US, tick-borne pathogens have become of increasing concern to scientists, medical professionals, and the public [8]. Along with *Borrelia burgdorferi*, blacklegged ticks can transmit disease agents such as *Anaplasma phagocytophilum* [9], *Babesia microti* [10], and *Ehrlichia muris eauclairensis* [11–13], the causative agents of anaplasmosis, babesiosis, and ehrlichiosis, respectively*.* Lone star ticks are associated with *E. chaffeensis* [14, 15] and *E. ewingii* [16], agents of ehrlichiosis, while winter ticks are associated with *A. marginale* [17, 18], which causes anaplasmosis in cattle. While deer keds are known to bite people and cause dermatitis in humans in Europe [19], they have generally been considered medically unimportant in North America because deer ked bites on humans are not widely reported in this region and evidence of pathogen transmission to humans is lacking [20]. Recent studies, including several from North America, have nevertheless identified zoonotic pathogens in deer keds collected from cervids; these include *Anaplasma* spp. [21–26], *Bartonella* spp. [23, 27–34], *Borrelia* spp. [24, 35], *Ehrlichia* spp. [35], and *Rickettsia* spp. [21, 23]. However, the role of deer keds in the transmission dynamics of these pathogens, if any, remains unclear.

Ectoparasites can acquire pathogens by feeding on infected hosts, but deer keds are one-host flies that are not known to interact with the primary reservoir hosts (i.e. small rodents) of many of these pathogens. As such, there is uncertainty as to how some of these typically tick-borne pathogens are acquired by deer keds. Vertical transmission of *Bartonella schoenbuchensis* by deer keds has been suggested, based on detection of the bacteria in immature stages and winged adults [23, 27, 31]. Another possible route is co-feeding transmission, whereby an uninfected deer ked could acquire a pathogen by feeding in close proximity to another infected ectoparasite, even if the host itself is not systemically infected with that pathogen. This route of pathogen acquisition is important in several *Ixodes* spp. tick-host systems [36–38].

This study was motivated by anecdotal reports that tick and deer ked infestations of white-tailed deer in the southeastern USA differ by body section and geographical location. Such spatial localization of different ectoparasite species on the host could have implications for disease dynamics, particularly if ticks and deer keds are sharing pathogens through co-feeding transmission. The probability of co-feeding transmission is known to decline with increasing distance between co-feeding ticks [39–42], so localization of ticks and deer keds on different parts of a host’s body would be expected to also reduce the risk of pathogen transmission between the two species.

Differences in on-host parasite distribution may reflect host suitability on a host landscape scale [43] as well as geographical region, ecological history, and competitive interactions. Previous studies have described feeding sites for *L. cervi* on red deer (*Cervus elaphus*), fallow deer (*Dama dama*), and moose in Europe [44, 45] and *I. scapularis* [46–50] and *A. americanum* [51] on white-tailed deer in North America, but these studies have been limited to a single geographical location. Competitive interactions between tick species have been demonstrated on white-tailed deer [50], but this has not been evaluated between two unrelated groups of ectoparasites, such as ticks and deer keds. Our objective was to evaluate the presence and on-host localization of tick and deer ked species on naturally infested white-tailed deer in different geographical regions. These data will help improve our understanding of parasite utility of the same host, which may influence pathogen sharing via co-feeding.

## Methods

**Fig. 1:**
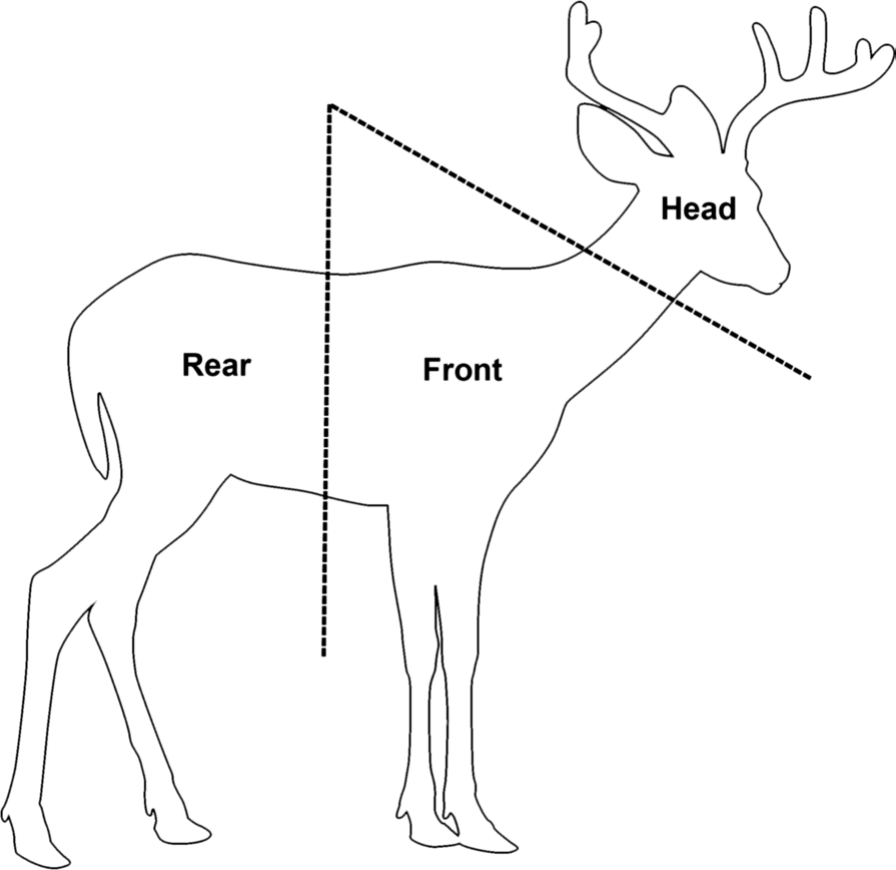
Diagram of the head (H), front (F), and rear (R) sections of white-tailed deer that were inspected for ectoparasites; each section was inspected for 2 min

During the 2018–2019 deer-hunting season, ectoparasites were collected from hunter-harvested white-tailed deer at check stations or meat processors in six eastern states (Table 1). Henceforth, “deer” will refer to white-tailed deer, unless otherwise stated. The inspection and collection process was reported in detail by Poh et al. [52]. In summary, the head, front, and rear of each deer (Fig. 1) were inspected prior to butchering, and ectoparasites were located visually and by running gloved hands or flea combs through the body hair. Ectoparasites found in each section were preserved in vials of 70% EtOH by body section.

**Table 1:** Locations where white-tailed deer were inspected for ectoparasites, ordered by decreasing latitude

Location	Sampling date	State	County	Latitude	Longitude	Site description
Bair’s Deer Processing	11/27–12/2/18	PA	Lancaster	40.1291	− 76.5853	Meat processor
Bennett Creek Conservation Park	12/20/18	MD	Montgomery	39.3062	− 77.2026	Multi-use park
Rachel Carson Conservation Park	12/17/18	MD	Montgomery	39.2199	− 77.0876	Multi-use park
Black Hill Regional Park	12/14/18	MD	Montgomery	39.1928	− 77.2975	Multi-use park
Woodstock Equestrian Park	1/4/19	MD	Montgomery	39.1884	− 77.4320	Multi-use park
Green Valley Processors	11/17/18	VA	Amhurst	37.5229	− 79.2916	Meat processor
H&M Processors	11/10/18	KY	Clay	37.1845	− 83.7640	Meat processor
Bethyl Valley, Oak Ridge	11/03/18	TN	Anderson	35.9656	− 84.2478	Agency check station
Oakmulgee, Talladega	11/23/18	AL	Hale	32.9565	− 87.4601	Agency check station

County and coordinates refer to the inspection site; some deer may have been harvested in adjacent counties

For collections made in Virginia, Kentucky, Tennessee, and Alabama, only deer freshly shot on the morning of collection (assessed by lack of rigor of the carcass) were inspected, and the three sections were inspected for 2 min each. Maryland conducted similar collections for deer freshly shot during county-run sharp shooting events, and each of the three sections were inspected for 2 min by two technicians for 4 person-minutes total per body section. In Pennsylvania, all available deer were inspected, and each section was inspected for 2 min by two individuals for 4 person-minutes total per body section. The maximum time since death for deer in Pennsylvania was 4 days, with most deer (71%) being checked the day after harvest and harvested deer held outside in low temperatures (< 4 °C) prior to inspection, which arrested arthropod movement (personal observation by KCP, JRE, MJS, ETM). While it is possible for deer keds to move or leave the host after host death, previous literature has described other dipteran species to slow or cease movement between 5 and 15 °C, depending on the species [53–56]. Additionally, since the methods of collection were the same within states, this would not be expected to affect relative abundances of different species or the patterns of abundances in different body sections within states. Methodological differences affecting ectoparasite abundance may be confounded with state, however, so we only compared abundances within each state.

Ticks were identified morphologically at the Hickling Laboratory at the University of Tennessee, the Penn State Veterinary Entomology Laboratory, and the Mullinax Spatial Wildlife Ecology Laboratory at the University of Maryland using standard keys [57–61]. Deer keds were identified at the Penn State Veterinary Entomology Laboratory using the key presented by Skvarla and Machtinger [5]. Deer keds and Pennsylvania tick vouchers were deposited in the Frost Entomological Museum at Penn State; Virginia, Kentucky, Tennessee, and Alabama tick specimens were retained in the Center for Wildlife Health at the University of Tennessee; Maryland tick samples were retained in the Mullinax Spatial Wildlife Ecology Laboratory at the University of Maryland. Henceforth, states will be referred to as the standard state abbreviations established by the US Postal Service [62].

### Statistical analyses

To test differences in abundances of ticks and deer keds within each body section per state, a Bayesian generalized mixed model was constructed using the package *brms* V.2.14.4 [63]. In the full model, the number of ectoparasites detected was modeled using a negative binomial distribution and contained the three-way interaction among ectoparasite species, state, and region along with a random effect of individual deer to control for repeated measures. All fixed effects had Student’s *t* priors, with three degrees of freedom, a mean of zero, and standard deviation of 10, while all other effects used the default priors [64]. The model was run for 10,000 iterations with a warmup of 2000. For model selection, all potential subsets of the full model that retained at least the random effect were compared with Pareto smoothed importance sampling leave-one-out cross-validation (PSIS-LOO [65]). A post-hoc analysis was conducted on the top model using Bayesian hypothesis tests for differences in abundance across body sections for each state and ectoparasite species.

## Results

A total of 1636 *I. scapularis* (adults), *D. albipictus* (nymphs and adults), and *A. americanum* (adults) ticks were collected from 168 deer across six states in the eastern US (Table 2). At least one tick species was collected from each state, with *I. scapularis* being the predominant species found in each state (Table 2). *Amblyomma americanum* and *D. albipictus* are included in summary statistics (Table 2), but were excluded from statistical analyses because of low sample sizes. Hereafter, “ticks” will refer to statistical results for *I. scapularis* only.

**Table 2:** Number of deer sampled and means (± SE) of deer keds and adult ticks collected from white-tailed deer in each state, November 2018–January 2019

State	Number of deer sampled	Deer ked^a^	*Ixodes scapularis*	*Amblyomma americanum*	*Dermacentor albipictus*
Alabama	14	3.00 (0.64)	2.43 (1.32)	0.07 (0.07)	0.00 (0.00)
Kentucky	20	0.00 (0.00)	13.80 (2.41)	0.00 (0.00)	0.75 (0.26)
Maryland	20	0.00 (0.00)	1.35 (0.33)	0.00 (0.00)	0.00 (0.00)
Pennsylvania	87	3.60 (0.49)	6.39 (0.79)	0.00 (0.00)	2.05 (0.49)
Tennessee	20	10.25 (1.73)	23.80 (2.79)	0.05 (0.05)	0.65 (0.38)
Virginia	7	0.00 (0.00)	8.29 (1.60)	0.00 (0.00)	0.14 (0.14)

^a^Deer ked species recovered were *Lipoptena cervi* and *L. mazamae*, which are geographically separate [5]. *Lipoptena cervi* were collected from PA only, and *L. mazamae* were collected from AL and TN

**Fig. 2:**
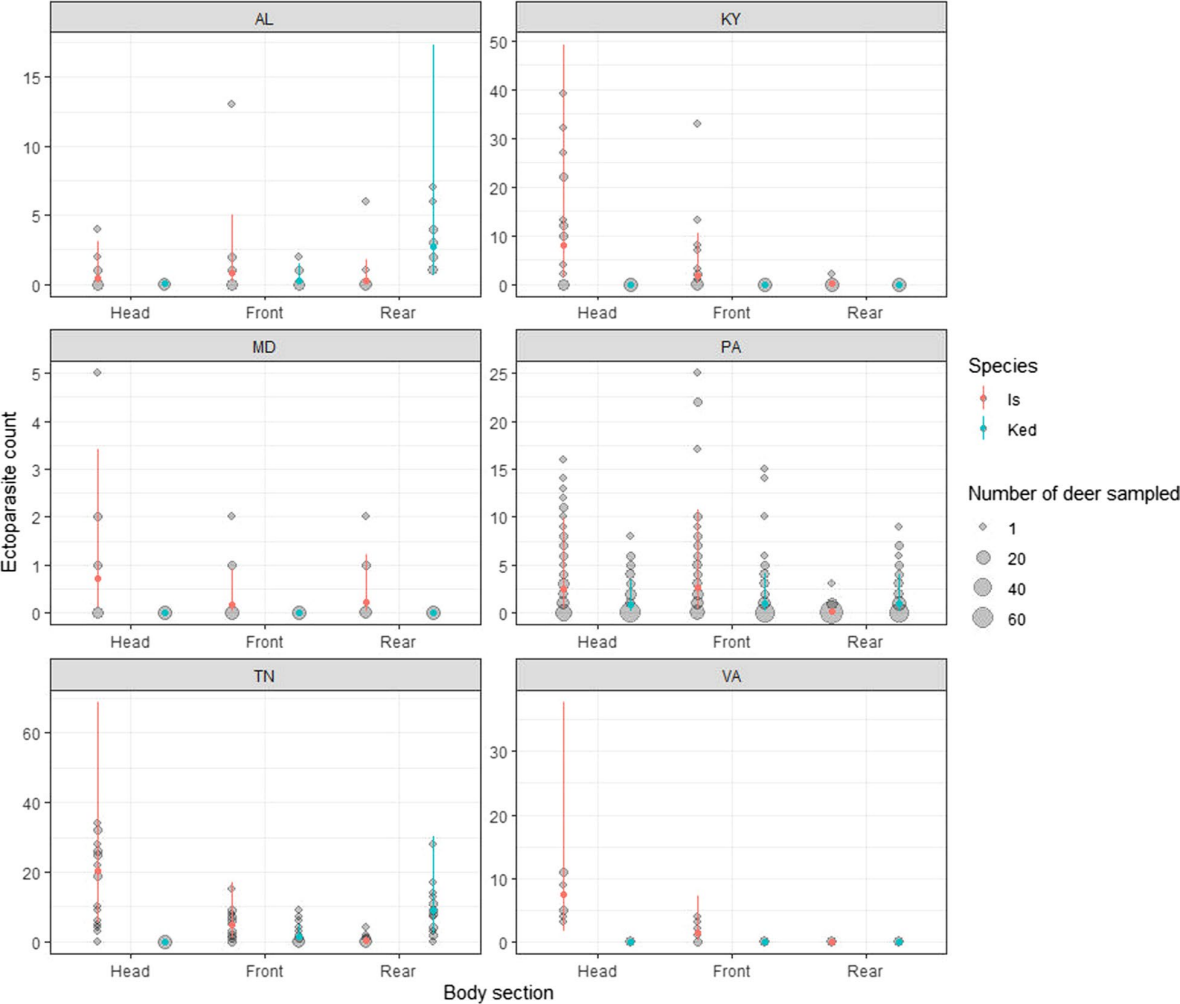
Predicted mean number of ticks and deer keds per deer body section (head, front, rear; ± 95% credible intervals, represented by vertical lines) expected to be found across body section and US state. Gray circles represent raw data (i.e. numbers of ectoparasites collected from each sampled deer)

The top model containing the three-way interaction among all effects performed best (Table 3), with clear variation across each variable and their interactions (Fig. 2, Table 4), and explained a reasonable proportion of the observed variance (*R*^2^ = 0.59 ± 0.04). The model showed good convergence between chains (Ȓ values ≤ 1.01; Table 4, Additional file 1: Fig. S1), unimodal posterior distributions, and good chain mixing. Ticks were consistently more common in the head section and less common in the rear section, with the exception of AL, where ticks were generally rare and no differences were detected (Fig. 2). Similarly, the only significant pairwise comparison in MD was between the head and front body sections, though data were limited in this state (Table 5).

**Table 3:** Model selection table using PSIS-LOO

Model	Δelpd	Δse	elpd loo	elpd loo se
~ State*Section*Species	0.00	0.00	− 1352.05	48.01
~ State*Section + State*Species + Section*Species	− 22.48	5.62	− 1374.53	48.35
~ Species*Section + State*Species	− 51.36	11.41	− 1403.40	48.66
~ State*Section + Section*Species	− 73.71	10.12	− 1425.75	50.31
~ State*Section + State*Species	− 97.70	13.03	− 1449.74	48.92
~ State + Section*Species	− 104.20	13.06	− 1456.24	50.27
~ Section*Species	− 119.56	13.15	− 1471.61	50.45
~ Section + State*Species	− 124.85	15.26	− 1476.89	49.06
~ State*Species	− 133.71	15.59	− 1485.75	49.20
~ State*Section + Species	− 141.87	13.88	− 1493.92	49.97
~ State*Section	− 159.35	14.82	− 1511.40	50.53
~ State + Section + Species	− 177.01	16.51	− 1529.05	50.61
~ State + Species	− 182.12	16.84	− 1534.16	50.63
~ State + Section	− 187.83	16.67	− 1539.88	50.80
~ Section + Species	− 200.39	17.72	− 1552.43	51.74
~ Species	− 205.62	17.89	− 1557.67	51.80
~ State	− 206.20	17.64	− 1558.24	51.38
~ Section	− 210.33	17.88	− 1562.38	51.87
~ 1	− 230.07	19.14	− 1582.12	52.94

elpd loo is the Bayesian leave-one-out cross-validation estimate of the expected log pointwise predictive density, Δelpd is the difference in the expected predictive accuracy for each model from the top model, and Δse is the standard error of this difference. All models with interactions also included all lower order effects

**Table 4:** Model effects summary table, with estimate, upper, and lower credibility intervals (CI) and Ȓ to assess chain convergence

Effect	Estimate	SE	Lower CI	Upper CI	Ȓ
Deer SD(intercept)	0.78	0.10	0.58	0.99	1.00
Intercept	0.85	0.17	0.51	1.20	1.00
StateAL	− 1.60	0.58	− 2.77	− 0.46	1.00
StateKY	1.21	0.39	0.46	1.99	1.00
StateMD	− 1.20	0.46	− 2.09	− 0.30	1.00
StateTN	2.14	0.36	1.44	2.86	1.00
StateVA	1.19	0.59	0.07	2.40	1.00
SectionFront	0.08	0.21	− 0.33	0.49	1.00
SectionRear	− 3.10	0.36	− 3.84	− 2.42	1.00
SpeciesKed	− 1.05	0.23	− 1.51	− 0.60	1.00
StateAL:SectionFront	0.47	0.73	− 0.94	1.91	1.00
StateKY:SectionFront	− 1.60	0.50	− 2.58	− 0.62	1.00
StateMD:SectionFront	− 1.61	0.76	− 3.15	− 0.18	1.00
StateTN:SectionFront	− 1.51	0.45	− 2.38	− 0.63	1.00
StateVA:SectionFront	− 1.83	0.79	− 3.37	− 0.30	1.00
StateAL:SectionRear	2.54	0.85	0.87	4.21	1.00
StateKY:SectionRear	− 1.95	0.99	− 4.05	− 0.17	1.00
StateMD:SectionRear	1.96	0.74	0.48	3.39	1.00
StateTN:SectionRear	− 0.82	0.64	− 2.07	0.43	1.00
StateVA:SectionRear	− 12.56	13.06	− 43.73	− 1.05	1.00
StateAL:SpeciesKed	− 5.46	3.65	− 14.55	− 0.54	1.00
StateKY:SpeciesKed	− 16.60	10.70	− 45.09	− 4.84	1.00
StateMD:SpeciesKed	− 14.66	10.35	− 40.97	− 2.61	1.00
StateTN:SpeciesKed	− 8.53	3.25	− 16.66	− 4.35	1.00
StateVA:SpeciesKed	− 15.66	12.33	− 47.21	− 3.28	1.00
SectionFront:SpeciesKed	0.09	0.32	− 0.54	0.73	1.00
SectionRear:SpeciesKed	3.24	0.44	2.41	4.11	1.00
StateAL:SectionFront:SpeciesKed	5.05	3.72	− 0.16	14.23	1.00
StateKY: SectionFront:SpeciesKed	− 4.51	12.32	− 33.31	14.03	1.00
StateMD: SectionFront:SpeciesKed	− 4.18	12.09	− 32.01	14.18	1.00
StateTN: SectionFront:SpeciesKed	8.25	3.29	3.91	16.44	1.00
StateVA: SectionFront:SpeciesKed	− 4.90	12.77	− 35.00	14.28	1.00
StateAL: SectionRear:SpeciesKed	5.63	3.71	0.43	14.82	1.00
StateKY: SectionRear:SpeciesKed	− 4.51	12.29	− 33.65	14.37	1.00
StateMD: SectionRear:SpeciesKed	− 5.82	12.13	− 34.78	12.49	1.00
StateTN: SectionRear:SpeciesKed	9.44	3.31	4.99	17.62	1.00
StateVA:SectionRear:SpeciesKed	− 2.58	13.01	− 32.46	18.91	1.00
Shape (phi)	0.79	0.10	0.62	1.00	1.00

**Table 5:** Post-hoc analysis of differences in abundance across body sections for each species state combination

State	Species	Contrast	Est	Error	Lower CI	Upper CI	Significance
AL	Is	Head—front	0.54	0.69	− 0.81	1.91	
AL	Is	Head—rear	− 0.58	0.77	− 2.10	0.92	
AL	Is	Front—rear	− 1.11	0.69	− 2.48	0.21	
KY	Is	Head—front	− 1.52	0.45	− 2.41	− 0.64	*
KY	Is	Head—rear	− 5.05	0.91	− 7.06	− 3.46	*
KY	Is	Front—rear	− 3.53	0.91	− 5.54	− 1.92	*
MD	Is	Head—front	− 1.53	0.74	− 3.04	− 0.16	*
MD	Is	Head—rear	− 1.14	0.65	− 2.45	0.10	
MD	Is	Front—rear	0.39	0.82	− 1.18	2.05	
PA	Is	Head—front	0.08	0.21	− 0.34	0.48	
PA	Is	Head—rear	− 3.11	0.36	− 3.84	− 2.44	*
PA	Is	Front—rear	− 3.19	0.36	− 3.92	− 2.51	*
TN	Is	Head—front	− 1.43	0.40	− 2.21	− 0.65	*
TN	Is	Head—rear	− 3.93	0.53	− 4.99	− 2.90	*
TN	Is	Front—rear	− 2.50	0.54	− 3.58	− 1.46	*
VA	Is	Head—front	− 1.74	0.76	− 3.23	− 0.26	*
VA	Is	Head—rear	− 15.39	11.16	− 44.86	− 4.15	*
VA	Is	Front—rear	− 13.65	11.18	− 43.06	− 2.37	*
AL	Lm	Head—front	5.77	3.82	0.69	14.70	*
AL	Lm	Head—rear	8.40	3.79	3.47	17.25	*
AL	Lm	Front—rear	2.62	0.75	1.23	4.17	*
PA	Lc	Head—front	0.17	0.24	− 0.31	0.65	
PA	Lc	Head—rear	0.14	0.25	− 0.34	0.62	
PA	Lc	Front—rear	− 0.03	0.24	− 0.50	0.44	
TN	Lm	Head—front	6.90	3.23	2.72	15.08	*
TN	Lm	Head—rear	8.75	3.22	4.60	16.91	*
TN	Lm	Front—rear	1.85	0.44	1.00	2.71	*

Contrasts for keds in states where they were never reported were removed. “Is” represents *Ixodes scapularis*, “Lm” represents *Lipoptena mazamae*, and “Lc” represents *Lipoptena cervi*

^*^Statistically significant at the 0.05 level based on 95% credibility intervals (CI) that do not cross 0

Deer keds were collected in three states: AL, PA, and TN (Table 2). Deer keds in AL and TN were identified as *L. mazamae*, whereas deer keds in PA were identified as *L. cervi*. *Lipoptena mazamae* from AL and TN were mostly confined to the rear body section and were significantly more abundant toward the rear (Table 5). In contrast, *L. cervi* in PA were evenly distributed across all body sections, with no significant difference in abundances among body sections (Table 5).

## Discussion

This study was motivated by anecdotal reports that tick and deer ked infestations of white-tailed deer differ by body section and geographical location. To quantify these reports, we collected ticks and deer keds from hunter-harvested deer brought to check stations or meat processors in six states in the eastern US during the fall 2018 hunting season. The results confirm that geographic location and co-occurring ectoparasite species impact the extent to which ticks and deer keds co-infest the same individual deer and the same body section of the host differs in ways that may be relevant to vector-borne disease ecology, such as co-feeding transmission as a method to share pathogens between two different ectoparasites.

Tick infestation of the deer we sampled was dominated by *I. scapularis*, primarily found in the head and front sections of the deer, regardless of state. Interestingly, previous reports of tick distributions on white-tailed deer in Pennsylvania recorded more *I. scapularis* on the body than the head during 3-min searches in two body sections [50], whereas in the current study *I. scapularis* was primarily found on the head and the front regions of deer. The deer tested in the previous study were confined to one fenced location, whereas the deer sampled in the current study were free ranging and from many areas of each state; we speculate that habitat structure may have played a role in these differences seen between studies. There are several reports of adult ticks attaching to the head or front sections of white-tailed deer, roe deer, and red deer [47–49, 66–68], perhaps because the head is the first part of the deer to encounter vegetation as it moves through the woods or during feeding [51]. If ticks are climbing onto the host from the legs, ticks may then migrate upwards to the front section because they detect exhaled carbon dioxide [69], which may further explain why ticks were mostly collected from the head and/or front sections. Greater hair density on the chest can protect ticks from desiccation, unfavorable temperatures, and removal by vegetation [66, 70], which may also explain this forward and upward movement and attachment. Furthermore, deer may be less able to effectively groom the head and front sections to dislodge fully-attached ticks.

It is unclear how adult deer keds find their hosts during the winged adult stage. There is evidence that deer keds typically use host movement as their primary cue to identify a host, although deer keds have occasionally been collected in traps baited with CO_2_ so they may also be attracted to exhaled carbon dioxide [71–75]. Relying on different cues during host-seeking and being able to navigate more quickly through pelage may be a proximate explanation for why deer keds differ from ticks in their localization on host deer.

The difference in spatial infestation patterns of northern and southern deer keds on cervid hosts suggests behavioral differences between the two deer ked species. *Lipoptena cervi*, collected in PA, are a non-native species that was first found in North America in 1907 and thought to have been introduced sometime in the late 1800s via deer imported from Europe [76, 77]. In contrast, *Lipoptena mazamae*, collected in TN and AL, are native to North, Central, and South America. Although *L. mazamae* is known as the “Neotropical” deer ked, Bequaert [78] reported this species of deer ked in the US, Mexico, Brazil, and Argentina. In this study, *L. mazamae* were more likely to be found in the rear section of white-tailed deer compared to the head or front sections, whereas *L. cervi* were spread more evenly across the body. In contrast, studies in Europe have found that *L. cervi* is more prevalent on the groin and flank of red deer and fallow deer and the front of moose [44, 45]. Host grooming response is a consideration, as deer keds have been found in the rumen of red deer and infested semi-domestic reindeer [*Rangifer tarandus tarandus* (Linnaeus, 1758)] and white-tailed deer show increased grooming behavior when deer keds are present [44, 79, 80], which suggests that deer can groom themselves (by licking, chewing, or scratching) to remove deer keds from their bodies. Additionally, deer ked bites produce papules that are accompanied by intense itching in humans [81]. Deer may also experience intense itching, thereby alerting deer to the presence of the deer ked followed by prompt removal through scratching, biting, and other grooming behaviors [44, 79, 80]. If deer are not able to reach their rear areas to groom themselves, this may be an advantageous location for deer keds. The native *L. mazamae* may have behaviorally adapted to deer grooming over evolutionary time whereas the non-native *L. cervi* do not have this behavioral response to native deer behavior or perhaps they have evolved to be more generalist in their feeding behavior and would not have the same response to deer grooming behavior.

Niche partitioning based on the coevolutionary relationship of deer keds with ticks may also explain differences in the number of ectoparasites found in each body section [82]. Native ticks and *L. mazamae* may have co-evolved to avoid resource competition, with Neotropical deer keds found at the rear and ticks found at the head. Niche partitioning has previously been shown for within-host parasite distribution with feather mites on birds, where different preferences and interactions (i.e. competition) between the mites on the bird host shaped the distribution of mites among and on individual hosts [83, 84]. On white-tailed deer, niche partitioning has been previously observed between *I. scapularis* and *D. albipictus*, with *D. albipictus* predominantly found in the head section when competing with *I. scapularis* [50]. As a recently introduced species, *L. cervi* may exhibit less niche partitioning and/or more competition with native ticks, which may explain why it is found more evenly over the entire body of the deer even when native ticks like *I. scapularis* are also present.

*Ixodes scapularis* and *L. cervi* feed on the same host in PA, where Lyme disease is endemic, which raises questions about the role deer keds could play in acquiring and potentially transmitting zoonotic pathogens now or in the future. Co-feeding ticks have been shown to horizontally transmit pathogens [37]. Deer keds feeding on deer could ingest pathogens from systemically infected deer or by co-feeding transmission from nearby ticks. For example, Matsumoto et al. [30] reported detection of *B. schoenbuchensis* in *L. cervi* and *I. scapularis* co-feeding on white-tailed deer. Because deer keds feed multiple times per day on a single animal and can live for at least a year and possibly longer on host [44, 76, 85], there could be multiple opportunities for tick-deer ked pathogen transmission. It remains unknown, however, whether deer keds that acquire a pathogen are capable of transmitting pathogens back to co-feeding ticks, humans, or other hosts [27, 31].

Based on findings in this study, which imply that ticks and Neotropical deer keds may not consistently feed in close proximity to each other, transmission of pathogens via co-feeding between ticks and deer keds may not be the primary method that deer keds acquire pathogens; deer keds may instead acquire these pathogens while feeding on an infected host or via vertical transmission. On the other hand, European deer keds may still participate in co-feeding transmission given that they were found in the same body sections as ticks. Clearly, the route of pathogen acquisition and potential transmission by deer keds warrant further future investigation. A study that looked in more detail at the spatial locations of tick and deer ked feeding sites on deer could help address this question, although one difficulty is that the long lifespan and high mobility of deer keds mean they could potentially acquire pathogens by co-feeding with ticks on a different host days to months before being collected.

The variability of deer ked and tick distributions on white-tailed deer highlights the need to further study deer ked-host and deer ked-tick relationships. Assuming white-tailed deer are reservoirs for pathogens that have been previously detected in *L. cervi* individuals, pathogens detected from deer keds would likely differ based on other ectoparasites and pathogens circulating in different geographical regions. A larger investigation spanning hosts from many states is necessary to improve our understanding of the potential role of deer keds in the transmission and survival of zoonotic pathogens. A temporal study of pathogens in ectoparasites feeding on the same deer would also help to understand the probability of co-occurrences of these pathogens in ticks and deer keds more generally.

## Conclusions

This is the first evidence of on-host spatial partitioning of ticks and two geographically distinct deer ked species (*L. cervi* in the northeast and *L. mazamae* in the southeast) on white-tailed deer hosts; partitioning was observed across multiple host body sections and across geographical regions. These differences in the distribution of ectoparasites on deer hosts could be related to the evolutionary relationship among ticks, deer keds, and their hosts, where native deer keds and ticks were more likely to confine themselves to specific sections of deer and native deer keds may be found in areas on the host that prevent premature removal via host grooming. *Lipoptena cervi*, which is not native to the US, was recovered in the northeast and was generally evenly distributed across all body sections of deer while *L. mazamae*, a native species, was confined to the rear sections. *Ixodes scapularis* was primarily found in the head or front sections in all cases. Given the spatial distribution differences of ticks and *L. mazamae* from white-tailed deer, co-feeding as a route of pathogen sharing between this species of deer keds and ticks may not be the most likely route of pathogen acquisition. Instead, deer keds may acquire pathogens when feeding on an infected host or through vertical transmission. However, finding *L. cervi* ubiquitously throughout all body sections of deer may support co-feeding transmission of pathogens with *I. scapularis*. Behavioral differences between related deer ked species emphasize the need to further investigate the relationship among deer keds, their hosts, and other ectoparasites and how these play a role in pathogen risk and transmission.

## Supplementary Information

**Additional file 1: Figure S1.** Rootogram showing performance of the model in the presence of zeroes in the dataset. The line and shaded area represent model predictions and 95% credible intervals. The histogram bars represent the actual data.

## Data Availability

The datasets generated and analyzed during the current study are available in the Penn State Data Archiving repository, https://data.psu.edu/.

## Abbreviations

AL: Alabama
EtOH: Ethanol
KY: Kentucky
MD: Maryland
PA: Pennsylvania
TN: Tennessee
VA: Virginia
