# Supplementary material for: Patterns of deer ked (Diptera: Hippoboscidae) and tick (Ixodida: Ixodidae) infestation on white-tailed deer (Odocoileus virginianus) in the eastern United States
Source: Parasit Vectors. 2022 Jan 20;15:31. doi: 10.1186/s13071-021-05148-9 (PMC8772158; doi:10.1186/s13071-021-05148-9)
Supplement: Supplementary file 1 — Additional file 1: Figure S1. Rootogram showing performance of the model in the presence of zeroes in the dataset. The line and shaded area represent model predictions and 95% credible intervals. The histogram bars represent the actual data. [file 13071_2021_5148_MOESM1_ESM.docx]

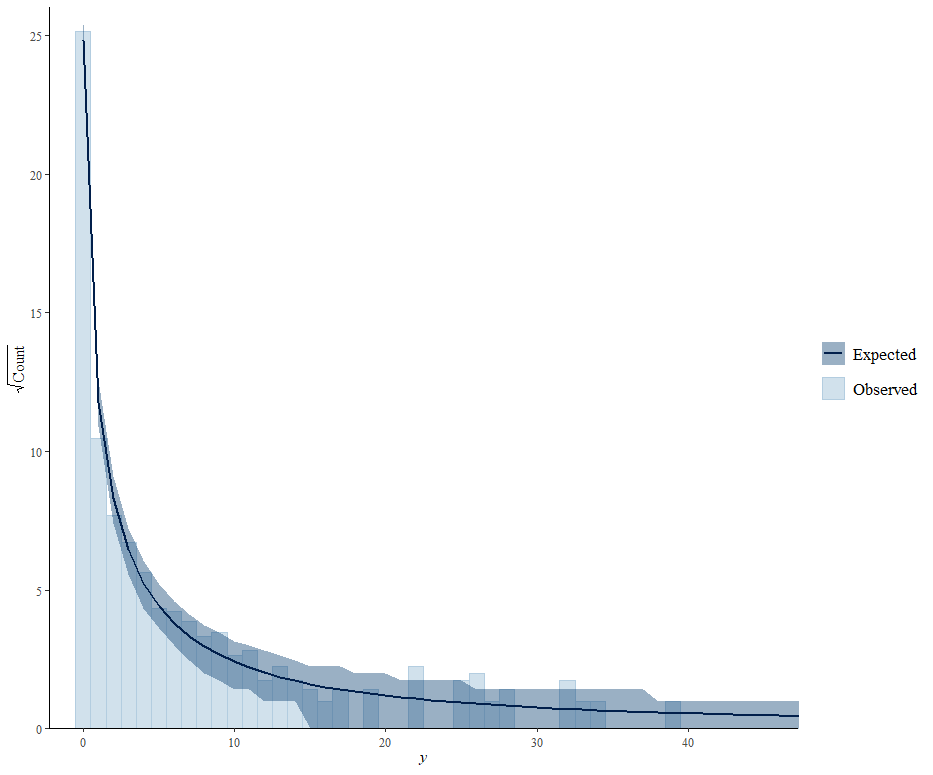
**Figure S1.** Rootogram showing performance of the model in the presence of zeroes in the dataset. The line and shaded area represent model predictions and 95% credible intervals. The histogram bars represent the actual data.
